# The influence of nevirapine and efavirenz-based anti-retroviral therapy on the pharmacokinetics of lumefantrine and anti-malarial dose recommendation in HIV-malaria co-treatment

**DOI:** 10.1186/s12936-015-0695-2

**Published:** 2015-04-25

**Authors:** Betty A Maganda, Eliford Ngaimisi, Appolinary AR Kamuhabwa, Eleni Aklillu, Omary MS Minzi

**Affiliations:** Department of Pharmaceutics, School of Pharmacy, Muhimbili University of Health and Allied Sciences, PO Box 65013, Dar es Salaam, Tanzania; Unit of Pharmacology and Therapeutics, School of Pharmacy, Muhimbili University of Health and Allied Sciences, PO Box 65013, Dar es Salaam, Tanzania; Division of Clinical Pharmacology, Karolinska Institutet, Karolinska University Hospital, C-168, SE- 141 86, Stockholm, Sweden

**Keywords:** Malaria-HIV, Efavirenz, Nevirapine, Lumefantrine, Drug-drug interactions

## Abstract

**Background:**

HIV-malaria co-infected patients in most parts of sub-Saharan Africa are treated with both artemether-lumefantrine (AL) and efavirenz (EFV) or nevirapine (NVP)-based antiretroviral therapy (ART). EFV, NVP, artemether and lumefantrine are substrates, inhibitors or inducers of CYP3A4 and CYP2B6, creating a potential for drug-drug interactions. The effect of EFV and/or NVP on lumefantrine pharmacokinetic profile among HIV-malaria co-infected patients on ART and treated with AL was investigated. Optimal lumefantrine dosage regimen for patients on EFV-based ART was determined by population pharmacokinetics and simulation.

**Methods:**

This was a non-randomized, open label, parallel, prospective cohort study in which 128, 66 and 75 HIV-malaria co-infected patients on NVP-based ART (NVP-arm), EFV-based ART (EFV-arm) and ART naïve (control-am) were enrolled, respectively. Patients were treated with AL and contributed sparse venous plasma samples. Pharmacokinetic analysis of lumefantrine was done using non-linear mixed effect modelling.

**Results:**

Of the evaluated models, a two-compartment pharmacokinetic model with first order absorption and lag-time described well lumefantrine plasma concentrations time profile. Patients in the EFV-arm but not in the NVP-arm had significantly lower lumefantrine bioavailability compared to that in the control-arm. Equally, 32% of patients in the EFV-arm had day-7 lumefantrine plasma concentrations below 280 ng/ml compared to only 4% in the control-arm and 3% in the NVP-arm. Upon *post hoc* simulation of lumefantrine exposure, patients in the EFV-arm had lower exposure (median (IQR)) compared to that in the control-arm; AUC_0-inf;_ was 303,130 (211,080–431,962) *versus* 784,830 (547,405–1,116,250); day-7 lumefantrine plasma concentrations was: 335.5 (215.8-519.5) *versus* 858.7 (562.3-1,333.8), respectively. The predictive model through simulation of lumefantrine exposure at different dosage regimen scenarios for patients on EFV-based ART, suggest that AL taken twice daily for five days using the current dose could improve lumefantrine exposure and consequently malaria treatment outcomes.

**Conclusions:**

Co-treatment of AL with EFV-based ART but not NVP-based ART significantly reduces lumefantrine bioavailability and consequently total exposure. To ensure adequate lumefantrine exposure and malaria treatment success in HIV-malaria co-infected patients on EFV-based ART, an extension of the duration of AL treatment to five days using the current dose is proposed.

## Background

Malaria and HIV/AIDS are highly prevalent in sub-Saharan Africa, accounting for almost 9% of the total disease burden and causing more than two million deaths each year [1-3]. In Tanzania, the prevalence of malaria and HIV/AIDS in adults (aged 15-49 years) is 5.3 and 5.1%, respectively [4]. Although, the epidemiology of HIV and malaria infections differs considerably, their geographical distributions do overlap, and co-infection is common among patients [2,3,5]. One of the critical areas of overlap between HIV/AIDS and malaria is the potentiality for drug-drug interactions (DDIs) between antiretroviral (ARVs) and anti-malarial drugs. Interactions between ARVs, such as non-nucleoside reverse transcriptase inhibitors (NNRTIs) or protease inhibitors (PIs) and anti-malarial drugs, especially those that are metabolized by cytochrome P450 enzymes (CYP450) are of particular concern [6-10].

Nevirapine (NVP) and efavirenz (EFV) has been the most widely used NNRTIs in the management of HIV infection particularly in resource-limited settings, including Tanzania [11,12]. However, due to the reported toxicities and ineffectiveness of NVP in the treatment of HIV-TB co-infected patients, NVP is no longer recommended as part of default regimen [11-14]. Based on World Health Organization (WHO) recommendation, currently, EFV-based antiretroviral therapy (ART) is a preferred option for initiating treatment in ART-naïve patients in most developing countries, including Tanzania and developed countries [11,13,14].

Equally, in all areas of malaria endemicity, WHO recommends the use of artemisinin-based combination therapy (ACT) as the first-line treatment for uncomplicated falciparum malaria [15]. Among ACT, artemether-lumefantrine (AL) is one of the most widely used drugs for the treatment of uncomplicated falciparum malaria in endemic countries [16].

Artemether, lumefantrine, EFV, and NVP are all metabolized by CYP450 enzyme systems, therefore predisposing them to possible DDIs. Artemether is metabolized to dihydroartemisinin (DHA) by CYP2B6, CYP3A4/5 and CYP2A6 [17]. DHA is rapidly inactivated to α-dihydroartemisinin- β glucuronide through glucuronidation via UDP-glucuronosyltransferases isoforms, UGT1A9 and UGT2B7 [18]. Lumefantrine is mainly metabolized by CYP3A4 to desbutyl-lumefantrine [19]. EFV and NVP are metabolized by CYP2B6 and CYP3A4, and are also potent inducers of these enzymes [20-23]. Drugs that induce or inhibit CYP450 enzymes may decrease or increase concentrations of concurrently administered drugs leading to treatment failure or drug toxicities, respectively [24]. So far, there is limited and inconclusive information on the potential interactions between anti-malarial drugs and NNRTIs [7-10].

Therapeutic efficacy of AL depends largely on the area under the plasma concentration time curve (AUC) above the minimum parasiticidal concentration of lumefantrine [19,25]. Day-7 lumefantrine plasma concentration is a surrogate marker of AUC [19]. Thus, any factor lowering day-7 lumefantrine plasma concentration could potentially increase the risk of treatment failure and emergence of drug resistance. Equally, EFV induction of CYP3A4 is influenced by CYP2B6*6 genotype, in a gene-dose dependent manner. Studies conducted in Tanzania show that allele frequency of CYP2B6*6 among Tanzanian are about 34-42% [26,27].

This study reports on the pharmacokinetic interaction between lumefantrine and EFV and/or NVP in HIV-infected patients with uncomplicated falciparum malaria, stable on ART. Equally, optimal lumefantrine dosage regimen for patients on EFV-based ART was determined using mathematical modelling.

## Methods

### Study design, subjects and ethical approval

This was a prospective, open label, parallel, non-randomized, pharmacokinetic drug interaction study with three arms. It was conducted at Bagamoyo District Hospital-HIV clinic in Tanzania between May 2010 and September 2012. HIV-1-infected patients with uncomplicated falciparum malaria were recruited. The study population was sub-grouped into three arms: patient taking 200 mg NVP twice daily (NVP-arm, n = 128) or 600 mg EFV once at night (EFV-arm, n = 66)-based ART for at least two months and those not yet on ART (control-arm, n = 75).

Patients were enrolled in the study if they met the following criteria: HIV-1 infection; age ≥18 years; auxiliary temperature ≥37.5°C or history of fever within 24 hours before visiting the clinic and with at least any of the following signs and symptoms: chills, sweats, headaches, muscle aches, nausea, vomiting, diarrhoea, body weakness, poor appetite, pallor, and enlarged spleen. Other patient-related parameters for inclusion in the study were haemoglobin (Hb) ≥7 g/dl; body weight ≥35 kg; and, microscopically confirmed *Plasmodium falciparum* infection.

The exclusion criteria included: signs of severe malaria; history of allergic reaction to any of the drug used in the study; evidence of chronic diseases such as renal and liver failure; use of anti-tuberculosis drugs for at least three months prior to enrolment; being on anti-malarial drugs four weeks prior to enrolment; being pregnant or nursing mother. In addition, use of alcohol, caffeine, drugs which induce or inhibit CYP3A4 and CYP2B6, prescription drugs, herbal medicines, oral contraceptives pills, grape fruits or juice was not permitted. Electrocardiogram, liver and kidney function tests, Hb test, blood smear for malaria parasite and pregnancy test for female patients were all performed prior to the enrolment in the study.

The study was approved by Muhimbili University of Health and Allied Sciences (MUHAS) Research and Ethics Committee. The study was conducted according to good clinical practice. The purpose of the study and its procedures were clearly explained to all study participants. A written informed consent was obtained from all participants prior to enrolment.

### Study procedures

#### Drug dosing, blood sampling and processing for pharmacokinetics

Patients meeting the inclusion criteria were enrolled and took the full dose (three-day course) of AL (Coartem® containing 80 mg artemether and 480 mg lumefantrine, Novartis Pharma AG, Basel, Switzerland) at 0, 8, 24, 36, 48, and 60 hours. The first and fifth doses of AL were administered under direct observed therapy (DOT) with 250 ml of milk (3.5% fat). The other four doses were taken at home. All patients were given verbal instructions on dosing intervals and on the importance of combining treatment with fatty meals. Additionally, patients were supplied with ten extra 250-ml packets of milk (3.5% fat) to be taken with the rest of the doses at home. Paracetamol was administered to all febrile patients. Patients were encouraged to return to the study site any time they felt ill. Patients who failed to return on the scheduled day were visited and assessed at home. If the study nurse failed to locate the patient’s house, they were classified as lost to follow-up. Any additional medications taken by patient during the study period were all documented in a case report form (CRF).

A pre-defined time schedule for blood sample collection was prepared and randomly assigned to patients. A maximum of six blood samples were collected from each patient over a 14-day time span. Emphasis was on the elimination phase, thus, collection of blood samples started after the fifth dose of AL administration. Prior to AL treatment 4, 2 and 1 ml of blood were collected in heparinized EDTA and in plain vacutainer tubes for lumefantrine, NVP-EFV and for clinical chemistry test, respectively. Just before taking the fifth dose of AL (48 hours), a cannula was inserted into a vein of the patient’s arm and 4 ml of blood sample was drawn for lumefantrine plasma level determination (trough concentration). After a patient has taken the fifth dose of AL, a maximum of three blood samples were collected from each patient at any of the following time points; 49, 50, 51, 52, 53, 54, 56, 58, 60, 72, 96, and 120 hours (sparse sampling). Similarly, all patients contributed blood samples on days 7 and 14 except for those who missed the scheduled visits.

The collected blood samples were kept in a cool box with an ice and were sent within 15 minutes to the laboratory at Ifakara Health Institute-Bagamoyo Research and Training Centre (IHI-BRTC) for centrifugation and storage. Blood samples were centrifuged (×2,000 g for 10 min) and plasma was transferred into the cryotube and stored in -80°C freezer. The plasma samples were transported on dry ice to MUHAS-Sida Bio-analytical laboratory in Dar es Salaam for analysis.

#### Drug analysis

All blood samples were analysed at MUHAS-Sida Bio-analytical laboratory. Lumefantrine plasma concentrations were quantified using high performance liquid chromatography (HPLC) method with UV detection as previously reported [28]. The coefficients of variation (CV %) during the analysis of lumefantrine was 2.5, 4.2 and 1.8% at 100, 1000, and 8,000 ng/ml, respectively. The observed accuracy during this analysis was -4.6, 3.6 and 7.4% at 100, 1000, and 8,000 ng/ml, respectively. The lower limit of quantification (LLOQ) for lumefantrine was 50 ng/ml. NVP and EFV were extracted from human plasma with protein precipitation procedure and were quantified using HPLC method with UV detection as earlier described, with minor modification [29]. The CV (%) during the analysis of EFV was 2.4, 0.7 and 1.4%, and for NVP was 2.1, 1.1 and 0.9% at 750, 5,000 and 12,500 ng/ml, respectively. The observed accuracy during the analysis of EFV was 5.2, 3.3 and 0.8% and for NVP was 5.5, 4.7and 2.4 at 750, 5,000 and 12,500 ng/ml, respectively. The lower limit of quantification for NVP and EFV was 250 ng/ml.

#### Adverse effects monitoring

Clinical chemistry tests and standardized questionnaires were all used to determine possible adverse effects of administered drugs on each visit.

### Pharmacokinetic and statistical analysis

#### Statistical analysis

Sociodemographic and clinical characteristics data were analysed using the Statistical Package for Social Sciences (SPSS) (version 16.0) software. Categorical variables were compared by Chi-square test. The one-way analysis of variance (ANOVA) test was used to compare the continuous variables between the three arms. Descriptive statistics were also used where appropriate. Baseline patient’s characteristics were summarized as medians with interquartile range (IQR) and means with standard deviation (SD). *Post hoc* pharmacokinetics parameters were expressed as medians (IQR) and statistical comparisons were done using one-way ANOVA after logarithmic transformation. A two-tailed P-value of <0.05 was considered statistically significant.

#### Pharmacokinetic analysis

Among the analysed samples, 17 (1.1%) displayed concentration below the LLOQ and were not used for population pharmacokinetic analysis. Lumefantrine compartmental kinetics was initially explored by plotting the plasma concentration time profiles on logarithmic scale. Models were fitted to the data through non-mixed effects modelling (NONMEM) (version 7.2, Icon Development Solutions) sub-routines ADVAN2 TRANS1 (one-compartment model) and ADVAN4 TRANS4 (two-compartment model). The pearl-speaks- NONMEM program (PSN version 3.7.5), R statistical program (version 3.0.1) and the Xpose program (version 4.4.0) were used for visual and quantitative diagnostics.

Different types of absorption models were tested including: zero order absorption, first order absorption with lag time and transit compartment absorption models (with fixed number of absorption compartments from one to ten). The pharmacokinetics parameters estimated in the final model were: clearance (CL/F), apparent volume of distribution in the central compartment (V_c_/F), peripheral apparent volume of distribution (V_p_/F), absorption rate constant (K_a_), inter-compartmental clearance (Q/F) and absorption lag time (tlag). All the parameter values, except K_a_ and tlag were standardized for total body weight using the allometric scaling formula; Tvθ = θ×[weight(kg)/70]^n^, where θ and Tvθ are typical parameter values for population and body weights, respectively; n is exponent of value ¾ for clearances and 1 for volumes [30].

Sensitivity/identifiability of model parameters was assessed by fixing the parameter values to double or half of those reported in the literature and rerunning the model fitting. A parameter was considered identifiable if such tweaking resulted in changes in objective function values (OFVs) for more than 3.84 units. Initial parameter values were obtained from a study conducted by Tarning *et al*. [31]. Since for oral kinetic data, bioavailability parameter (F) is usually unidentifiable, its value was fixed to 1 (100% bioavailability) to allow accurate estimation of other parameters. Between subject and between occasions variation (random effects) for all the fixed effect parameters was tested. Exponential models were assumed for all the random effects. Sequential stochastic model building procedure was used, whereby one random effect parameter was evaluated at a time. A combined (additive and proportional) error model was used to account for residual variation in the measured lumefantrine concentrations. A better model was chosen based on likelihood ratio test by using the OFVs computed by NONMEM program. For a hierarchical model, a decrease in OFVs by more than 3.84 units (equivalent to P value <0.05) was considered significant improvement to model fit. The first-order conditional estimation (FOCE) of pharmacokinetic parameters was used throughout the modelling.

The full covariate model building approach was used so as to decide which parameter-covariate relationships were statistically and clinically significant [32,33]. Initial parameter covariate relationships were chosen based on exploratory graphics (random effects *versus* covariate plots) and mechanistic plausibility of parameter-covariate relationship. Since ART can influence clearance and bioavailability of lumefantrine simultaneously, thus, type of concomitant ART were included as covariate on both parameters in the full model. No other parameter-covariate relationships were included. The estimated fixed effect parameters and their covariance (uncertainty) were used to simulate fixed effect parameter values for 1,000 patients. The resulting posterior distribution of the parameters (mean and 95% confidence interval) relative to the reference values (population typical values) was used to decide on the clinical and statistical significance of the parameter-covariate relationship. Finally, the full model was reduced to the final model by removing relationships that were not statistically significant or clinically important.

Precision of the model parameters was obtained through the NONMEM covariance functionality. Evaluation of the final model was done using goodness-of-fit plots, visual predictive check (VPC) and confidence intervals (CI) or relative standard errors (RSE) of the estimated parameters.

#### Pharmacokinetic simulations and post hoc calculations for AUC and C_max_

The final model parameters (including random effects) were used to simulate pharmacokinetic parameters AUC_(0-inf)_, C_max_, T_max_ and day-7 lumefantrine plasma concentrations at different dosage scenarios: 480 mg twice daily for three days (normal dosing); five or seven days; and, 1,200 mg twice daily for three days. In each scenario, 9,960 stochastic simulations were made. The obtained AUC_(0-inf)_, C_max_, T_max_, and day-7 lumefantrine plasma concentrations were compared between the arms through visual inspection of box plots, summary statistics and ANOVA after logarithmic transformations.

## Results

### Baseline characteristics and treatment outcomes

A total of 269 HIV-infected patients with uncomplicated falciparum malaria were enrolled. No significant difference was observed between patients’ baseline characteristics for the three arms studied, except in the case of patients’ age (P = 0.015), Hb (P = 0.036) and CD4+ cell counts (P = 0.002) (Table 1). The results for malaria treatment outcomes have been reported elsewhere [34]. AL was well tolerated in all enrolled patients; the observed and reported drug side effects were mostly mild in severity.

**Table 1:** **Patients baseline characteristics**

**Parameters**	**ARMS**			
	**Control (n=75)**	**Nevirapine (n=128)**	**Efavirenz (n=66)**	**P value**
Sex (female) %	65	79.5	52.3	
Median age in years	38 (19–64)	42 (21–67)	43 (39–66)	0.015
Temperature mean, SD ± OC	38.1 ± 0.8	37.8 ± 1.3	38.3± 0.9	0.485
Median weight (IQR)	56 (41–92)	55 (41–78)	58 (36–84)	0.953
Median (range) parasite density/μL	1280 (560–4040)	4040 (600–261520)	3440 (480–126960)	0.564
Haemaglobin (g/dL) median (IQR)	13.9 (12.2-15.2)	12.1 (11.2-13.5)	12.3 (10.2-13.6)	0.036
CD4+ count (x10^6^/L) median (IQR)	402 (66–964)	354 (19–1781)	298 (9–694)	0.002

### Pharmacokinetics of lumefantrine

**Figure 1:**
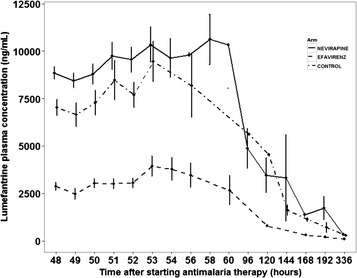
Lumefantrine concentration time profiles for HIV-malaria-co-infected- patients on antiretroviral drugs and those not yet on antiretroviral drugs.

A total of 1,514 venous plasma samples were available for analysis and comparison. Lumefantrine plasma concentration time profile for the three arms studied is presented in Figure 1. Visual inspection of the concentration-time plot on a logarithmic scale indicated a two-compartment pharmacokinetics. Data for this study was well described by two-compartment pharmacokinetic model with first order absorption and lag-time.

**Figure 2:**
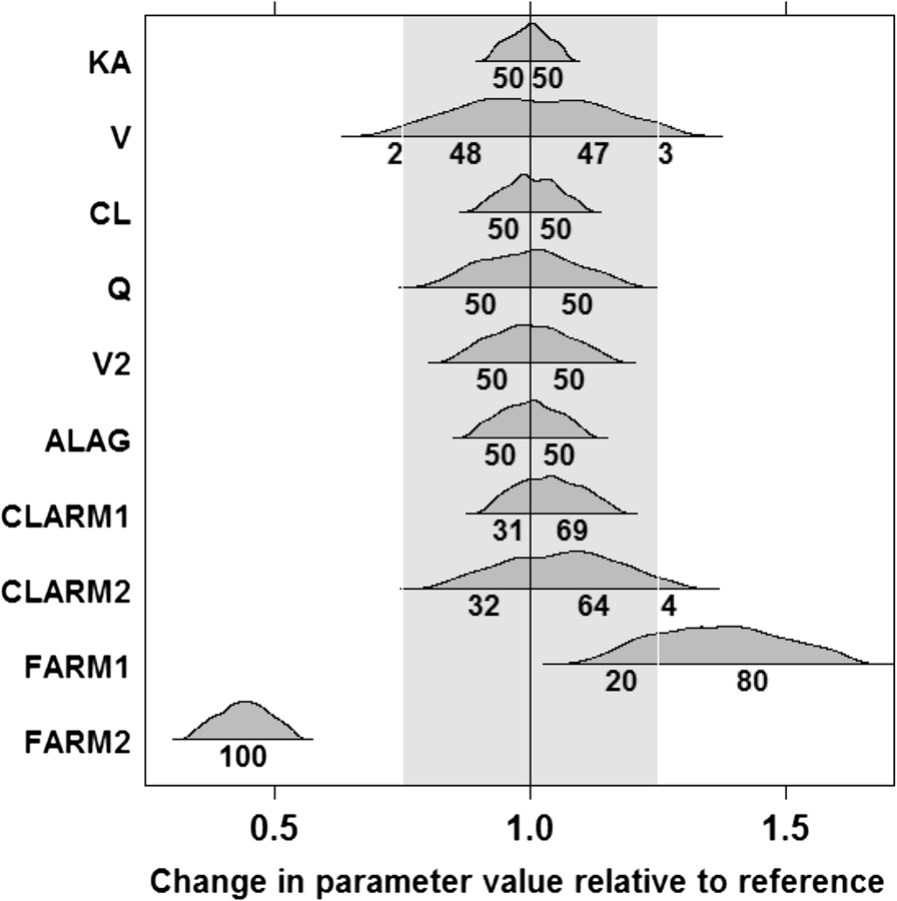
Estimated parameters and covariate effects along with uncertainty relative to the reference value. The light grey shaded region represents clinically irrelevant effect (+/- 20%) region. Numbers outside the shared region represent the approximate probability of the effect to be clinically relevant. Numbers inside the region represent the probability distribution within the clinically irrelevant region on either side of the reference line. FARM1 = Bioavailability for NVP-arm; FARM2 = Bioavailability for EFV-arm; CLARM1 = clearance for NVP-arm; CLARM2 = clearance for EFV-arm.

The full covariate model building approach identified concomitant EFV and NVP as statistically significant covariates on bioavailability but not on clearance (Figure 2). It can be implied from the full model that EFV co-treatment causes clinically important decrease in lumefantrine bioavailability. Although NVP is shown to increase lumefantrine bioavailability, data were insufficient to conclude the clinical importance of this effect.

**Figure 3:**
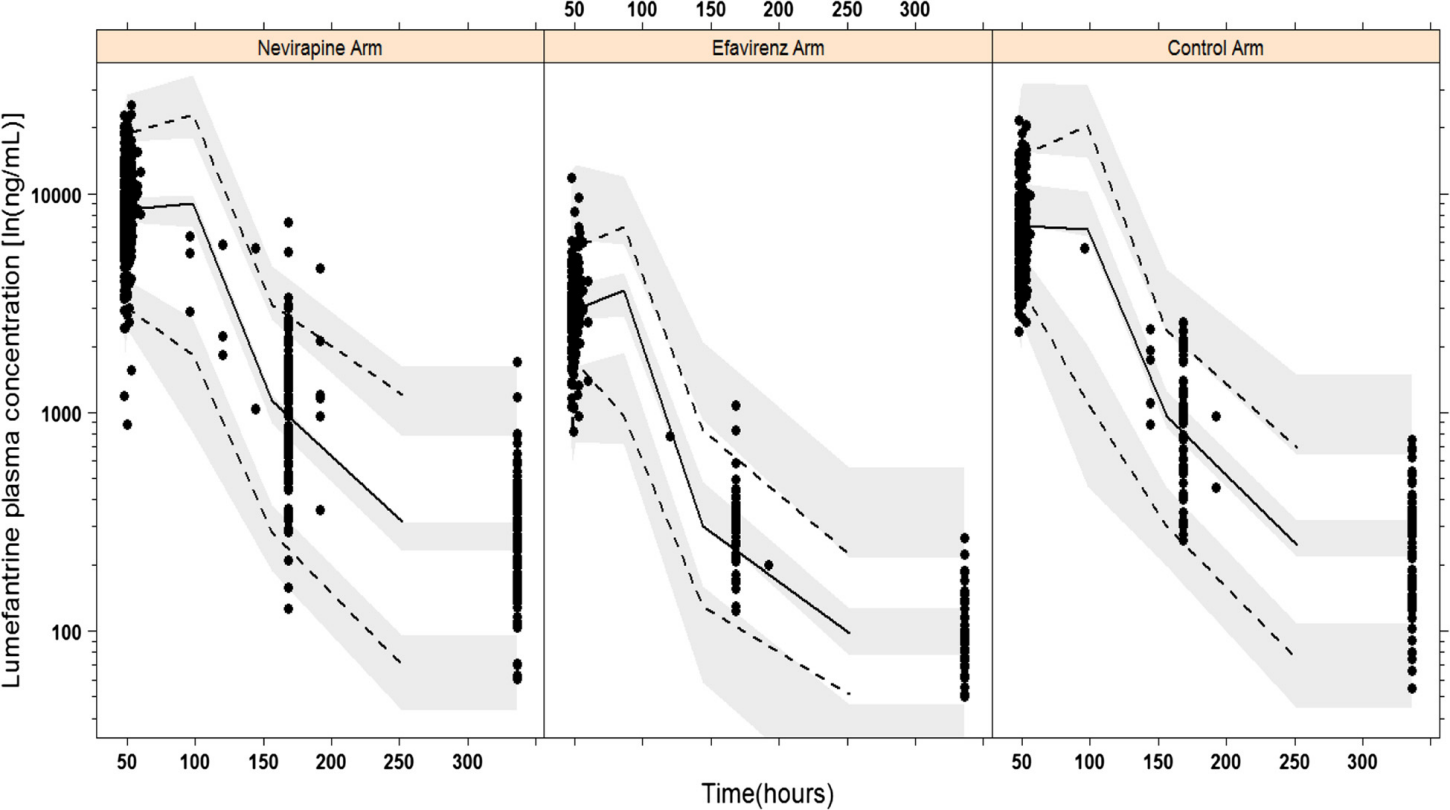
Visual predictive checks of the final two-compartment-model describing the population pharmacokinetics of lumefantrine. The aforementioned visual predictive checks are for HIV-malaria co-infected patients on NVP-based ART (NVP-arm), EFV-based ART (EFV-arm) and those not yet on antiretroviral therapy (control-arm) treated with artmether-lumefantrine.

EFV and NVP co-treatment as important covariates on the bioavailability of lumefantrine and other final model pharmacokinetics parameter estimates are indicated in Table 2. Evaluations of the final model did not show any model misspecification. The computed η-shrinkage was high for most of the computed pharmacokinetics parameters (defined as ≥30%), except for Q/F (23%) and F (5%) (Table 2). The performance of the final model was highly predictive as enlightened by the VPC plots (Figure 3).

**Table 2:** **Parameter estimates describing lumefantrine population pharmacokinetics for HIV-malaria co-infected patients treated with artemether-lumefantrine**

**Parameters**	**Description**	**Estimates (95% CI)**	**BSV (RSE %) (Shrinkage %)**	**BOV (RSE %) (Shrinkage %)**
Ka (hr-1)	Absorption rate constant	0.032 (0.029-0.034)	29% (18) (30)	
Vc/F (liters)	Central volume	25.6 (16.21-34.99)	82% (24) (51)	
CL/F (liters/hr)	Clearance	4.54 (3.913-5.167)	0*	19% (22) (44)
Q/F (liters/hr)	Inter-compartmental exchange Clearance	1.23 (0.99-1.47)	27% (24) (23)	
Vp/F (liters)	Peripheral Volume	203 (167.13-238.87)	39% (22) (37)	
t lag (hr)	Absorption lag time	1.45 (1.25-1.65 )	0*	
F1	Bioavailability (population typical value)	1*	47% (11) (5)	
F1 EFV	Relative bioavailability for patients on Efavirenz	0.42 (0.34-0.5)		
F1 NEV	Relative bioavailability for patients on Nevirapine	1.32 (1.08-1.52)		
ADD	Additive residual error	26.30 (4.15-48.44)		
PROP	Proportion residual error	0.083 (0.06-0.10)		

RSE, relative standard error; CI, confidence intervals; BSV, Between Subject Variability; BOV, Between Occasion Variability; *,Fixed to this value.

### Pharmacokinetics of lumefantrine in patients treated with EFV-based ART

The raw data indicated that 32 and 4% of patients in the EFV-arm and in the control-arm, respectively, had day-7 lumefantrine plasma concentrations below the suggested cut-off value of 280 ng/ml. The simulated data indicated that, at the current AL dosing, the pharmacokinetic parameters AUC_0-inf_ and C_max_ were lower by 61.3 and 61.1%, respectively, compared with that in the control-arm (Table 3). No statistical significant difference was observed between the T_max_ of this arm and that of the control-arm (P = 0.86). Day-7 lumefantrine median (IQR) plasma concentrations were also 60.9% lower compared with that in the control-arm (P < 0.001) (Table 3).

**Table 3:** **Simulated lumefantrine pharmacokinetics parameters in HIV-malaria co-infected patients treated with AL (9960 simulations)**

**Parameters**	**Control-arm**	**Nevirapine-arm**	**Efavirenz-arm**			
Dose	480 mg bid 3 days	480 mg bid 3 days	480 mg bid 3 days	480 mg bid 5 days	480 mg bid 7 days	1200 mg bid 3 days
C_max_ (ng/ml)	8192.7 (5664.3 - 11896.8)	10229 (7173.4 - 14606)	3182.2 (2198.4 - 4586.1)	3678.2 (2609.7 - 5150.7)	3887.5 (2690.4 - 5531.4)	7955 (5496.2 - 11464)
AUC_0-inf_ (ng.hr/ml)	784830 (547405–1116250)	977645 (688477–1383975)	303130 (211080–431962)	513760 (359212.5 - 713715)	755090 (528277–1086525)	757835 (527702–1079925)
T_max_ (hr)	66.1 (63.6 - 67.7 )	66.1 (60.0 - 67.7 )	66.1 (60.4 - 67.6)	-	-	-
Simulated day 7 plasma concentration (ng/ml)	858.7 (562.3 - 1333.8)	1090.3 (704.4 - 1680.4)	335.5 (215.8 - 519.5)	1039.4 (678.1- 1552.8)	1079.2 (694.1- 1689.6)	838.9 (539.6 - 1298.9)
Observed day 7 plasma concentration (ng/ml)	970 (562.1 - 1729)	1125 (638.8 - 1913)	300.4 (220.8 - 343.1)	-	-	-

AL, artemether-lumefantrine; bid, after every 12hrs; AUC_0-inf_, plasma AUC from 0 hour extrapolated to infinity; C_max,_ maximum plasma concentration; T_max_, time to reach maximum plasma concentrations. The presented values above are expressed as median with inter-quartile range.

### Pharmacokinetics of lumefantrine in patients treated with NVP-based ART

In the present study, only 3% of patients in the NVP-arm had day-7 lumefantrine plasma concentration below the therapeutic cut-off point of 280 ng/ml. The simulated data indicated that, at the current AL dosing, AUC_0-inf_ and C_max_ were higher by 24.6 and 24.8%, respectively, in patients on NVP-based ART compared with that in the control-arm (Table 3). There was non-significant difference in the day-7 lumefantrine median (IQR) plasma concentrations between the NVP-arm and control-arm (P = 0.063). Likewise, no statistical significant difference was observed between the T_max_ of this arm and that in the control-arm (P = 0.86) (Table 3).

### Dose regimen simulations for patients on EFV-based ART

Extending the duration of treatment of AL from three to five days using the current dose taken twice daily, the simulated AUC_0-inf_ and C_max_ were slightly lower compared to that observed in the control-arm. Nonetheless, the simulated day-7 lumefantrine plasma concentrations in the EFV-arm were comparable to that observed in the control-arm.

**Figure 4:**
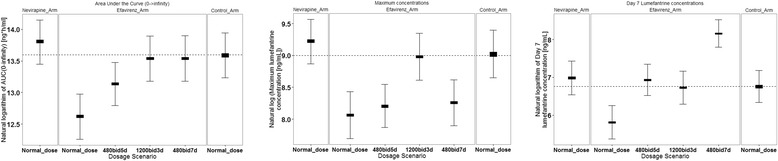
Simulated lumefantrine pharmacokinetic parameters at different dosage scenarios for HIV-malaria-co-infected- patients on efavirenz-based treatment. The box and whiskers represent 95% confidence interval of the mean and the first and third quartiles of the simulated.

Equally, when the duration of treatment of AL was extended from three to seven days while using the current dose and taken twice daily or escalation of lumefantrine dose to 1,200 mg (2.5 times the normal dose) taken twice daily for three days, the simulated AUC_0-inf_ and C_max_ and day-7 lumefantrine plasma concentrations were comparable to that observed in the control-arm. These results are summarized in Table 3 and Figure 4.

## Discussion

A fixed-dose combination of ACT, such as AL, is most widely used in the treatment of uncomplicated and multidrug-resistant falciparum malaria in areas of endemicity, including Tanzania [16,35,36]. AL has gained its popularity as the drug of choice in malaria-endemic countries due to its safety and efficacy [37,38]. Few studies have reported on the potential effect of NNRTIs on the pharmacokinetics profile of AL in either HIV-infected patients or healthy volunteers without malaria [7-10]. The present study evaluated the pharmacokinetics profile of lumefantrine in HIV-infected patients on ART and those not yet on ART with uncomplicated falciparum malaria treated with AL.

The major finding from this study is the significant reduction of lumefantrine bioavailability, exposure and day-7 plasma concentrations among patients on EFV-based ART compared to ART naïve patients. On the contrary, lumefantrine bioavailability, total exposure, and day-7 plasma concentrations were higher among patients on NVP-based ART compared to ART naïve patients, although, this differences did not reach statistical significance.

The observed results in EFV-treated patients are in agreement with results reported from studies conducted in Uganda and USA [7,10]. However, some differences were observed from Huang *et al*. study which could be accounted for by sample size, age, sex, disease status (healthy *versus* HIV-malaria co-infection), ethnicity, and genetic. The observed results in the EFV-arm appear to be mediated through a pharmacokinetics interaction between lumefantrine and EFV, via induction of either gastrointestinal or hepatic CYP3A4 or both. The induction of CYP3A4 by EFV is reported to be concentration and time dependent [22,39,40], and is via the human nuclear pregnane X receptor (hPXR) and the human constitutive androstane receptor (hCAR) [22,23]. Transactivation of hCAR and hPXR receptors in return increases the functional activity of CYP3A4 [22,23]. Thus, the persistent induction of CYP3A4 by EFV could explain the low lumefantrine exposure in the EFV-arm [39,40]. Accordingly, the EFV-arm results are in keeping with what has been reported by others [41,42].

On the other hand, the findings obtained in the NVP-arm are consistent with the results from a South African study [9], but different from a Ugandan study [10] in which lumefantrine day-7 plasma concentrations and exposure were decreased, although the decrease was not statistically significant. Study design, food intake, patient’s immune status, patient’s body weight, smoking, alcohol intake and disease status (HIV only *versus* HIV-malaria co-infection) might have contributed to the observed differences. Lumefantrine AUC increases with subsequent dose and its absorption is enhanced by food intake, particularly fat meal, just as small as 1.2 g of fat is enough to increase the absorption of lumefantrine to about 16-fold [43]. In the present study, all patients were supplied with full cream milk (3.5 g fat) and had to take the latter before dosing with AL (all doses). In the Ugandan study patients were asked to take the five doses of AL (among the six doses of AL) at home while encouraged to take each dose with milk, only the last dose was given with fatty meal at the clinic [10]. In addition, the Ugandan study enrolled only those patients with CD4 cell count of ≤200 cells/cu mm, while in the present study participants in the NVP-arm had a median CD4 cell counts of >350 cell/cu mm [10]. Thus, the differences in immune status among patients involved in the two studies could be accounting for the observed differences. Patients with low CD4 cell counts are often sick and frail with reduced food intake, hence compromising AL absorption.

Although NVP and EFV are reported to be both an inducer of CYP3A4 and CYP2B6, the induction capacity is reported to be disproportional [22,23]. EFV is reported to be a five times more potent inducer of CYP3A4 than NVP at a given drug concentration [23]. The differences in the induction capacity of CYP3A4 enzyme might explain the observed differences in the reduction of lumefantrine bioavailability, exposure and day-7 plasma concentrations by the two drugs. Similarly, Mouly *et al.* reported non-inducibility of CYP3A4 by NVP. HIV-infected patients on ART were involved [44]. Further research exploring the possible mechanism of interaction between NVP and lumefantrine is highly required, as this study was not designed to explore the aforementioned interactions.

The objective of using anti-malarial drugs for treatment of uncomplicated malaria is to clear all parasites from the body, thus cure the infection [19]. The AUC of lumefantrine above the minimum parasiticidal concentration is the determinant of treatment response in patients with uncomplicated malaria and reflects the degree of exposure of parasite to lumefantrine after artemether is cleared [19,25]. Accordingly, low lumefantrine exposure is associated with increased risk of malaria treatment failure and emergence of drug-resistant parasites [19,45].

The results of the present study are in agreement with a recent published study which reported high rate of parasitaemia recurrence in HIV-malaria co-infected patients on EFV-based ART as compared to those on NVP-based ART or not yet on ART and treated with AL [34]. This observation is of concern, especially with the escalated use of EFV in HIV-infected patients in Tanzania, where malaria is also endemic [11]. On the other hand, EFV induction of CYP3A4 is influenced by CYP2B6*6 genotype, in a gene-dose dependent manner [39,40]. Previous studies have indicated that allele frequency of CYP2B6*6 among Tanzanian is about 34-42 [26,27]. This calls for a continued usage of NVP among HIV-infected patients (without potential fatal side effects) in malaria-endemic areas, since phasing out this ARV may create challenges in the management of uncomplicated malaria in this population. Alternatively, based on our finding, increasing the duration of malaria treatment in patients receiving EFV-based ART may salvage the risk of sub-therapeutic plasma exposure of lumefantrine and hence, treatment failure.

A new dosage regimen of AL that would achieve the therapeutic efficacy in patients on EFV-based ART with uncomplicated falciparum malaria was simulated using the final predictive model. The predictive model through simulation suggested that escalating lumefantrine dose to 2.5 times the normal dose taken twice daily for three days would achieve the targeted therapeutic plasma concentration in this population comparable to that in the control-arm. Importantly, in a recent published study an increment of lumefantrine dose to 250% was proposed for malaria patients co-treated with EFV-based ART [46]. Nonetheless, lumefantrine absorption is dose-dependent and its oral absorption decrease as doses (amount) increases. It has been also reported that lumefantrine absorption is close to saturation at the current doses in the standard regimen [47]. Thus, any increase in lumefantrine doses may result into kinetics shifting from first order to zero order absorption rate leading to unexpected under-dosing of patients*.*

The predictive model also suggested that increasing the duration of treatment from three to five days or seven days would achieve the targeted lumefantrine exposure and day-7 plasma concentrations above the minimum parasiticidal concentration [25]. Similarly, in a study from Thailand, increase of AL duration of treatment from 60 to 96 hours, resulted into an increase of lumefantrine exposure from 60 to 100%, respectively [48]. However, increasing the duration of AL treatment to five or seven days may be associated with reduced adherence and increased risk of adverse events. Thus, achieving maximum malaria cure rate and treatment adherence, a twice daily dose of AL given for five days may be more appropriate than the seven days for this population. Likewise, Tarning *et al.* in their study suggested the same for pregnant women with day-7 lumefantrine plasma concentration below the therapeutic cut-off value of 280 ng/ml [31].

Although, predictive models used in drug dosage regimen optimization may provide necessary information with respect to quantitative understanding of dose exposure-response relationship, whilst, accounting for patients’ behaviour such as adherence, nevertheless, this may not be achieved *in vivo* due to non-linear relationship between PK/PD, drug toxicity and poor adherence to treatment*.* Thus, patient’s adherence to treatment and safety need to be evaluated, before implementation of the proposed dosage regimen.

The final model described well the present data and is highly predictive; the computed results for the fixed and random effects are highly informative and robust. The reported high η-shrinkage in this study could be contributed by the sparseness of sampling [49].

## Conclusion

Pharmacokinetics of lumefantrine in the present study was best described with two-compartment models with first order absorption and lag time. Co-administration of AL with EFV-based ART but not NVP-based ART significantly reduces lumefantrine bioavailability and consequently total exposure. Results from the predictive model suggested that; extending the duration of AL treatment from three to five days using the current dose taken twice daily will be adequate for lumefantrine exposure and treatment success in HIV-infected patients with uncomplicated falciparum malaria on EFV-based ART. In addition, the observed low lumefantrine exposure in HIV-malaria co-infected patients on EFV-based ART poses a significant challenge in treating malaria in this population. Therefore, in malaria- and HIV-endemic areas where AL is widely used, clinicians may be required to undertake a thorough assessment of patient’s eligibility for ART initiation, before choosing an appropriate NNRTI for initiation, as NVP may still be an appropriate alternative.
